# Changes in Ground Reaction Forces and Center of Pressure Parameters of Paws When Wearing Dog Boots in Dogs

**DOI:** 10.3389/fvets.2022.906277

**Published:** 2022-07-12

**Authors:** Bianca Bieber, Bianca Reicher, Alexander Tichy, Barbara Bockstahler

**Affiliations:** ^1^Section of Physical Therapy, Small Animal Surgery, Department of Companion Animals and Horses, University Clinic for Small Animals, University of Veterinary Medicine, Vienna, Austria; ^2^Department of Biomedical Sciences, Bioinformatics and Biostatistics Platform, University of Veterinary Medicine Vienna, Vienna, Austria

**Keywords:** dog, boots, gait analysis, center of pressure, ground reaction forces

## Abstract

Dog boots are commonly used as protective footwear against snow, ice, hot sand, road salt, and paw injury. Only a few studies exist in veterinary medicine that capture the impact of dog boot replacements, such as bandages, on ground reaction forces (GRF) in dogs. To our knowledge, no studies have investigated the effect of dog boots on the center of pressure (COP) in dogs. This study investigated changes in the GRF of the whole limb and selected COP parameters of the paws while wearing dog boots in five Labrador Retrievers. After habituation, data were collected by walking and trotting dogs over a pressure platform without boots (control measurement) and under five different test conditions (wearing boots on all limbs, boots on both front limbs, boots on both hind limbs, one boot on the left front limb, and one boot on the right hind limb). The most prominent change was detectable when one boot was worn on the left front limb, with a decrease of peak vertical force (PFz%) in the left front limb at trot which led to a significant difference between both front limbs and a significant increase of PFz (%) in the right hind limb. Additionally, in both tempi, the vertical impulse (IFz%) showed significant differences between the front limbs; in trot, there was also an increase in the right front limb compared with the control. Furthermore, some significant changes in COP parameters were detected; for instance, all test conditions showed a significant increase in COP area (%) at the right front limb during walking compared to the control. Therefore, our results show that wearing the tested dog boots in different constellations seems to have an impact on GRF and some COP parameters.

## Introduction

Dog paws are exposed to great stress depending on the animal's use and habitat. They have functional footpads on each of the four weight-bearing toes and a central pad centrally located in the area of the distal metapodium. These hairless, heavily keratinized pads with subcutaneous fat pads have a cushioning effect and are exposed to friction. Contextual separation of pads, such as ulcers, penetrating wounds, abrasions, and chemical or thermal injuries are quite common and often need to be treated surgically or with bandages (1). Injuries to the paws of working and sporting dogs are among the most common (2–4). Dog boots can fulfill a protective function and reduce the number of paw injuries (5). As these are worn for longer periods of time, it is important to ensure the physiological loading of the extremities during the gait cycle. In rehabilitation, especially in the case of neurodegenerative diseases such as degenerative myelopathy, a chronic progressive nerve demyelination that can lead to paraparesis of the hindquarters, paw boots can be used for abrasion protection in addition to physiotherapeutic measures such as training on an underwater treadmill and passive movement exercises (6). Since dog boots are frequently used in everyday life, it is important to understand their influence on dog gait and load distribution.

Several methods are applicable for studying motion sequences. One of them is the measurement of the so-called ground reaction forces, where participants walk and trot over force (7, 8) or pressure plates (9, 10). Force plates directly measure the acting forces in newtons (N), and these generated forces describe the summation of those that act on the limbs during the stance phase, and are divided into vertical, craniocaudal, and mediolateral forces. Pressure plates are used to determine the pressure in Newton/cm^2^. From the pressure data obtained, the acting forces can then be calculated by multiplication of the used area and expressed in Newton, however, only those forces acting in the vertical direction are recorded by pressure measuring plates. Because vertical forces have the largest amplitude (11) they are most frequently used in research and both systems can be used to describe GRF in sound and orthopedically diseased dogs.

In addition to the evaluation of GRF, these gait measurement systems also allow a description of the pressure distribution within the paw as well as the measurement of the center of pressure (COP). The COP describes the point at which the current GRF vector acts and can be described for the whole body as well as for the limbs. If observed during walking or standing (statokinesiogram), a constant change in its position over time creates a COP path. Its course can be described by different COP parameters, such as craniocaudal and mediolateral COP excursions, the path length, velocity as well as the COP area. Measurements of the COP can be used to describe biomechanical adaptations in response to neurological (12) and orthopedic (13, 14) conditions in humans. In veterinary medicine, it has been shown in dogs that the COP can be successfully used to investigate dogs with neurological disorders (15), to detect lameness and describe paw dynamics (16–19). Using static post-urography Manera et al. (16) found out that in lame dogs COP parameters were altered in the statokinesiogram and stabilogram. Also Carillo et al. (17) used these methods in a sample of dogs with elbow dysplasia and cranial cruciate ligament rupture, demonstrating a higher COP sway, or “instability,” in lame dogs. Lopez et al. (18) used the limb center of pressure to examine if differences between lame and non-lame limbs in dogs with elbow dysplasia were detectable. The results showed, among other things, that due to a shortened swing phase, the limb COP is shortened and cranialized in the lame limb if compared to the non-lame limb. In a recent study, COP data were collected for all four limbs in 24 dogs with cubarthrosis and 19 with coxarthrosis, then compared with 20 orthopedically healthy dogs. Dogs with cubarthrosis showed an increase in craniocaudal COP excursion (%) of the lame limb and an increase in mediolateral COP excursion (%) of the ipsilateral hind limbs. Furthermore, the COP area (%) increased in both the hind limbs. The main change observed in the coxarthrosis group was an increase in the mediolateral COP excursion (%) and COP area (%) in both hind limbs (19).

In human medicine, the effect of different types of footwear on GRF has been successfully investigated. For example, a recent study investigated the GRF during barefoot walking and wearing of sandals, flip-flops, and trainers in 10 men with no history of distal extremity orthopedic disorders using a force plate. The investigation showed a significantly lower stance phase duration when barefoot compared to all types of shoes studied. Furthermore, there was a flatter increase in the loading rate of the 1st peak vertical GRF of the trainers compared to when barefoot, or wearing sandals and flip-flops. The authors concluded that this was due to the thicker and cushioned sole of sports shoes (20).

In another human medical study, in which special pressure sensors were attached to the plantar foot surface of the subjects or in the shoe insoles, a significant difference was detected between barefoot and shoe-wearing subjects in the measured plantar pressure and the pressure contact area. For instance, compared with people wearing shoes, a higher mean pressure and smaller contact area with the ground was measured in the area of the heel when wearing no shoes (21).

In contrast to human medicine, in veterinary research, only a few studies have addressed special devices on dog paws. A recent study investigated the effect of dog boots on GRF by mimicking paw boots with ethylene vinyl acetate pads attached to all paws of six beagles. After a short familiarization period, they were trotted over a force plate. No significant differences in stance phase duration, vertical impulse, and maximum vertical force were found between the measurements with and without dog boots. However, there was a greater increase in the force-time curve to PFz (peak vertical instantaneous loading rate) in shod dogs (*P* < 0.05). The authors concluded that dog boots can definitely fulfill a protective function against environmental influences; however, a variance in the load when wearing dog boots can possibly result in overstressing of the surrounding tissue (5).

In addition to the successful use of pressure plates to measure gait analysis in both healthy and lame dogs and cats (17, 22–24) and objective measurement of the therapeutic success after surgical interventions (25, 26), the measurement of the effectiveness of canine paw devices on GRF, such as ToeGrips® (27, 28), has been used in veterinary medicine. In both ToeGrips® studies, rubber rings were attached to the claws of the weight-bearing toes of orthopedically healthy dogs and the dogs were then walked over a pressure plate after a short acclimatization period. In the first study, there was a significant reduction in PFz in both hind limbs; in the second study, there was only a tendency to be detected. Similarly, the first study measured the prolongation of SPD in all limbs and reported an increase in IFz in both front limbs and the right hind limb. In the second study, there was a reduction seen in IFz for both hind limbs and no significant change was observed in SPD.

No study to date has investigated the effects of dog boots on GRF. This study was carried out using commercially available dog boots^1^.

The hypothesis of this study was that despite previous habituation, wearing dog boots on one or more limbs leads to detectable changes in ground reaction forces and selected COP parameters in dogs' limbs.

## Materials and Methods

### Ethics

All measured data were obtained from voluntary sound participants using the same standardized measurement procedure. All measurements were discussed and approved by the Institutional Ethics and Animal Welfare Committee in accordance with the Good Scientific Practice guidelines and national legislation (ETK-103/06/2019).

### Dogs and Inclusion Criteria

This paper is an extract from a diploma thesis (29), in which the ground reaction forces of five sound Labrador Retrievers were measured. All dogs were female with a mean age of 4.6 ± 2.3 years and a mean body mass of 26.27 ± 3.1 kg.

Each dog underwent an orthopedic and neurological examination according to Baumgartner (30) at the facilities of the Section for Physical Therapy and Rehabilitation of the University of Veterinary Medicine Vienna to rule out an undiagnosed musculoskeletal disorder. Only dogs with unremarkable orthopedic and neurological examinations, with measured limb loading within the norm (symmetry indices SI <3 %, see below), were included in the study.

### Pawz® Rubber Dog Boots

**Figure 1:**
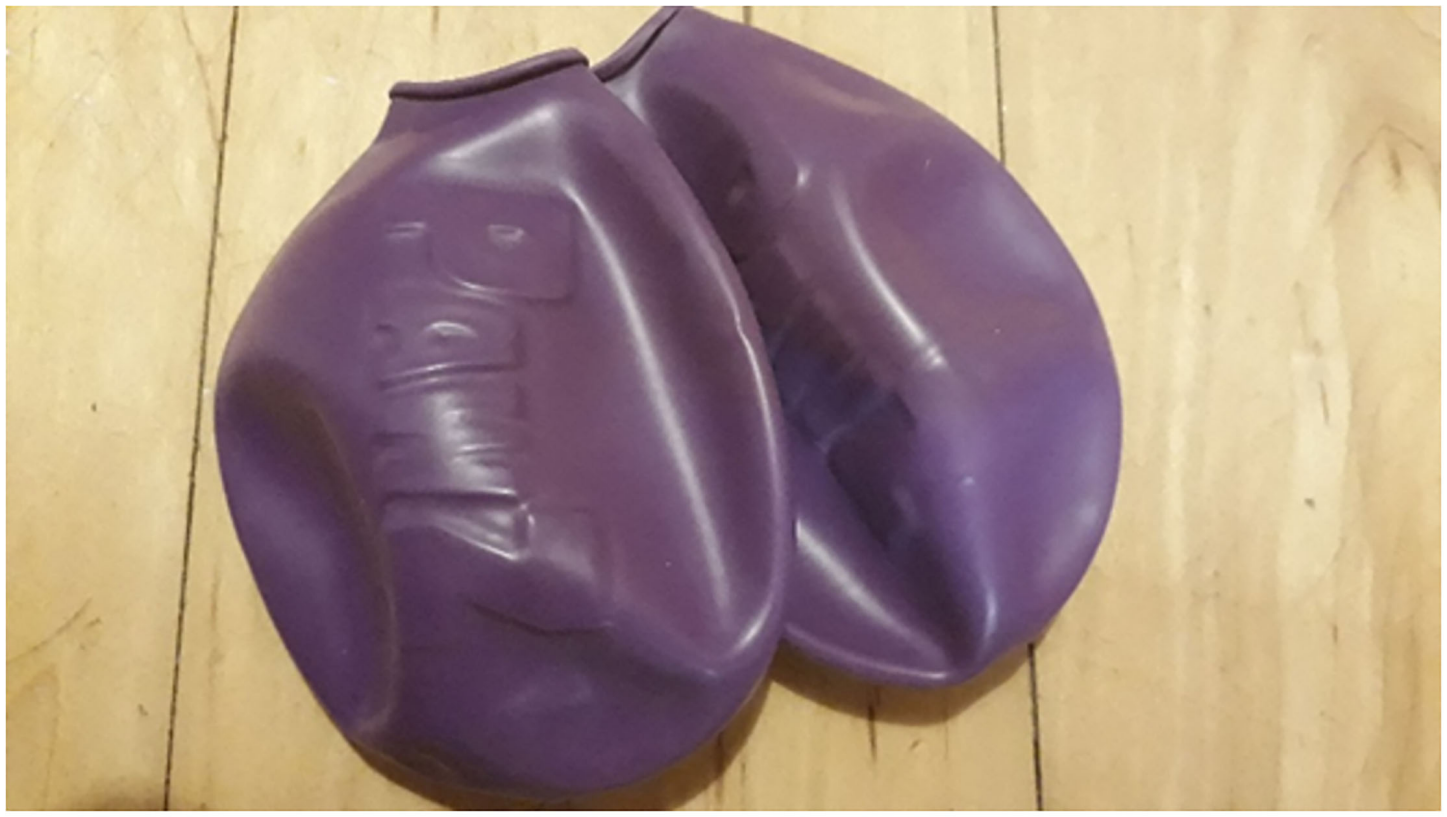
The tested dog boots in size “large” (see text footnote 1).

The used dog boots^1^ are paw-protection boots that are available in seven sizes and made out of rubber. According to the manufacturer's instructions, shoes were fitted by measuring the distance from the most caudal point of the metacarpal or metatarsal pad to the tip of the longest claw. Figure 1 shows the used large dog boots.

### Equipment

The pressure plate used (FDM Type 2, Zebris Medical GmbH, Allgäu, Germany) measured 203 × 54.2 cm and is able to detect the pressure of the dog's paws using 15,360 piezoelectric sensors at a sampling rate of 100 Hz. In order to ensure unhindered movement of the dog and handler, the pressure plate was surrounded by chipboard and a plain area was prepared and covered with a 1 mm thick, black, non-slip rubber mat, made out of polyvinylchlorid. To assign the measured values to the correct limb of the dog during data evaluation, each measurement was filmed with a Panasonic camera (model NV-MX500).

### Measurement Procedure

To participate in this study, all the participants were required to attend the facility twice. At the first appointment, orthopedic and neurological examinations were performed. Then, the dog and owner were given time to familiarize themselves with the movement analysis laboratory and the pressure measurement plate. The procedure was explained, and a written declaration of consent to participate in the study was obtained.

Subsequently, the first GRF measurement was performed. Each dog walked and trotted over a pressure plate without booting. For each measurement, the dog was walked/trotted over the pressure plate until a minimum of 5 valid steps were collected. Only steps during which the dog carried its head straight and walked at a steady pace were considered valid. The difference in velocity at which the dogs crossed the plate should be within a range of ±0.3 m/s at a walk (31), a maximum of 0.5 m/s at a trot (32) and an acceleration of ±0.5 m/s^2^.

This procedure was followed for all the subsequent measurements. Symmetry indices (SI) were calculated as described below (see the investigated parameters) to determine whether the dog met all inclusion criteria.

The owner of each dog received four boots of appropriate size. The dogs were then given at least 1 week to become accustomed to wearing the boots in their everyday environment. The owners were instructed to train their dogs under all conditions described below that they would be facing during the subsequent measurements. The dog should wear the boots according to each of the planned measurement conditions for a few minutes, but no longer than 15 min at a time, over the course of a week.

On the day of the actual trials, six measurements were performed during walking and trotting. First, another measurement without boots was performed to ensure that the participant still met the inclusion criterion of SI <3%. The results of these measurements also served as controls for all test conditions.

These test conditions consisted of five different combinations of the number and placement of the worn boots on

- all four limbs
- both front limbs
- both hind limbs
- the left front limb
- the right hind limb

To prevent falsification due to a habituation effect, measurements for the five test conditions were conducted in a randomized order for each animal. The participants were always given a 5–10 min long break, during which they were accustomed to the condition of the following measurement. All data were analyzed using Pressure Analyzer 4.3.2.0 software (Michael Schwanda, Königstetten, Austria) and then exported to Microsoft® Excel® 2016.

### Investigated Parameters

#### GRF Parameters

The peak vertical force (PFz, N) and vertical impulse (IFz, Ns) of each limb were normalized and given as a percentage of the total force [PFz (%), IFz (%)]. The formula is given here based on an example for the calculation of PFz of the left front limbs:


(1) $$PF_{\text{zFL}}\;\left(\%\right)=100\;x\;\frac{\left(PFzFL\right)}{\left(PFzFL+\;PFzFR+PFzHR+PFzHL\right)}$$

Where PF_zFL/FR_ = maximal vertical force front left/front right and PF_zHR/HL_ maximal vertical force hind left/hind right.

For both parameters, a symmetry index was calculated to describe the percent degree of deviation from symmetry in the front and hind limbs (10). The formula is given here based on an example for the calculation of IFz modified from Budsberg et al. (33):


(2) $$\text{SI IFz }(\%)=abs\;x\;\left(\;\frac{(IFzl\;-\;IFzr)\;}{\left(IFzl\;+\;IFzr\right)}\;\right)x\;100$$

Where SI_IFz_ = symmetry index of the vertical impulse of a limb pair, IFz_l_ = Vertical impulse of the left forelimb or hindlimb, lFz_r =_ vertical impulse of the right forelimb or hindlimb, abs = absolute.

The stand phase duration (SPD) was further investigated. It describes the period of time during which the paw contacts the ground and is given as a percentage of the total SPD of all four legs. The other parameters under investigation were speed (m/s), stride length (m), and paw contact area (cm^2^).

#### COP Parameters

**Figure 2:**
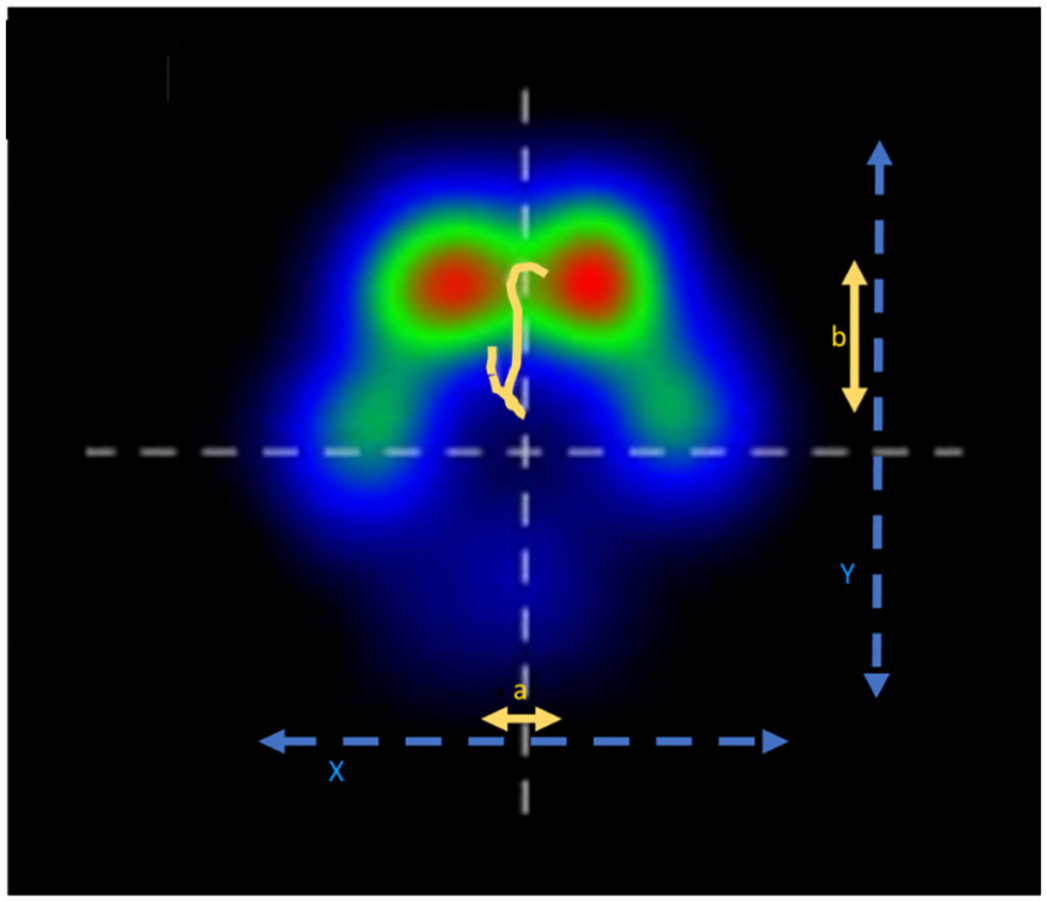
Paw contact area and COP path, a: medio-lateral COP displacement, b: cranio-caudal COP displacement, X: maximum width of paw contact area, Y: maximum length of paw contact area.

The center of pressure (COP) describes the point at which the current GRF vector acts. If it is observed during walking, a constant change in its position during contact with the ground creates a COP path (19). As shown in Figure 2, the mediolateral and craniocaudal COP displacements represent the difference between the maximum positive and negative excursions along the craniocaudal and mediolateral axes. They were expressed as a percentage of the maximum width or length of the paw contact area. The COP area, which includes all points of the COP, was normalized to the paw contact area and expressed as a percentage using the following formula:


(3) Area (%)=100/mean A ×COP area

Where A = mean paw contact area of a leg in mm^2^ and COP area = COP area of the respective leg in mm^2^.

### Statistical Analysis

All the parameters were evaluated using a linear mixed model. The Shapiro–Wilk test was used to check the assumption of a normal distribution of the data. Different conditions and limbs were included as factors in the model. *Post-hoc* testing with Sidak's alpha error correction was performed to compare the control measurements under different conditions. Analyses were performed using IBM SPSS v24 software. For each comparison, *P* ≤ 0.05 was considered significant.

## Results

The stride length (m) and velocity (m/s) showed no significant changes between each test condition and the control during walking and trotting.

**Figure 3:**
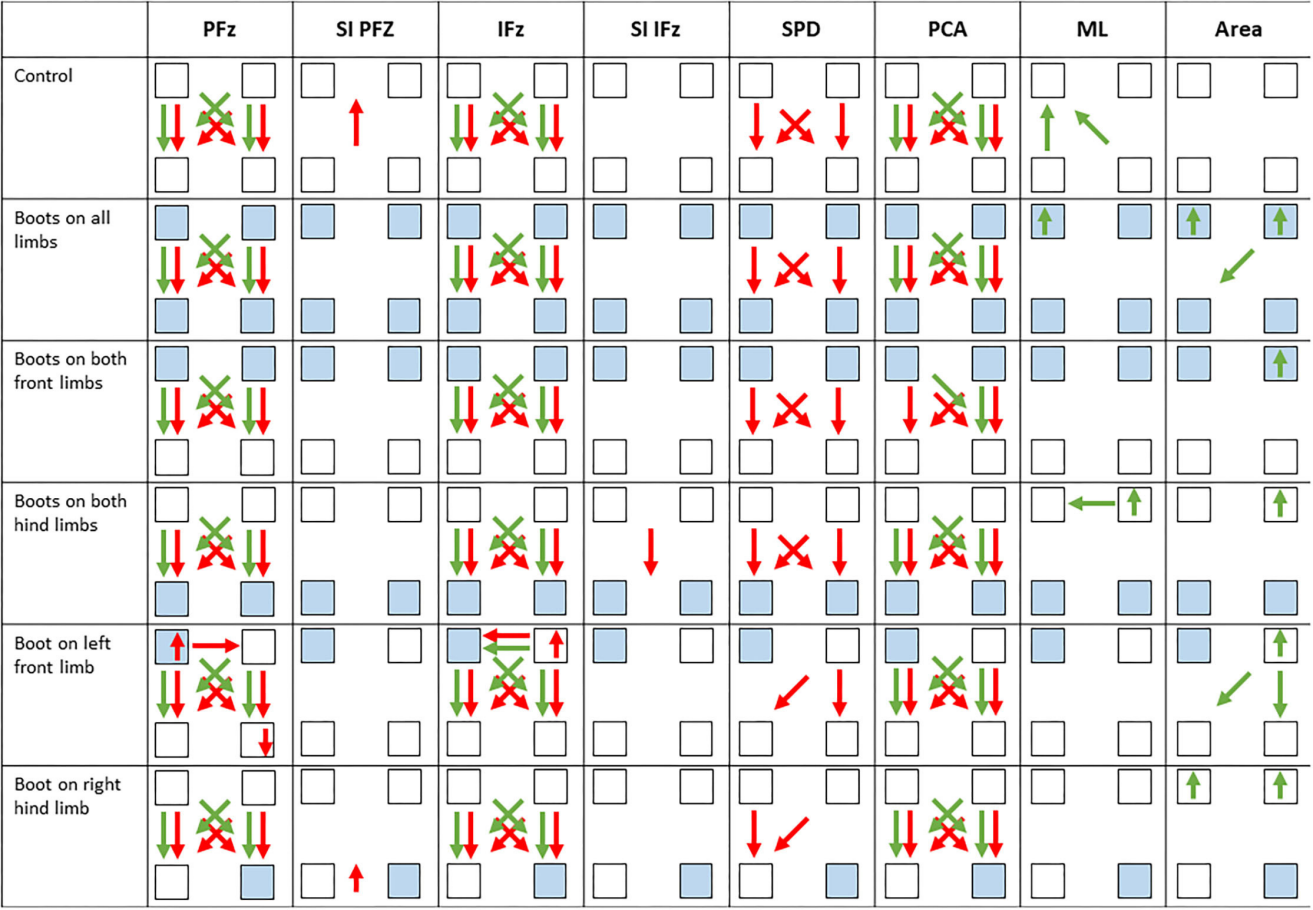
Visualized overview of altered parameters, based on at least five steps/dog per measurement. Data were evaluated using a linear mixed model and *post-hoc* testing with Sidak's alpha error correction. Each square represents a limb where those in blue represent boot-wearing limbs. The arrows between the squares show significant differences between the legs where the green represents walking and red for trotting. The arrowhead points in the direction of the lower value in each case. In case of a significant difference between the control and a test condition, an arrow within or beside the square indicates an increase or decrease of the value. For instance, when a dog boot was worn on the left front limb, PFz (%) at walk showed higher values in both front limbs and a decrease in the left front limb and an increase in the right hind limb compared to the control. Further, a significant difference between both front limbs was observed. PFz, peak vertical force; SI PFz, symmetry index of PFz; IFz, vertical impulse; SI IFz, symmetry index of IFz; SPD, stance phase duration; PCA, paw contact area; ML, medio-lateral COP displacement; area, COP-area.

An overview of all mean ± standard deviation, where significant differences between the control and test conditions are marked with superscript symbols, is given in Tables 1–**6**. Figure 3 shows a visualized overview of all significantly changed parameters.

**Table 1:** Mean ± standard deviation of all parameters for the condition “no dog boots.”

	**Limb**	**PFz (%)**	**SI PFz (%)**	**IFz (%)**	**SI IFz (%)**	**SPD (%)**	**SL (m)**	**PCA (cm^**2**^)**	**v (m/s)**	**COP cran-caud (%)**	**COP med-lat (%)**	**COP area (%)**
Walk	LF	30.45 ± 1.81^*^, ^‡^	1.12 ± 0.96	31.64 ± 0.46^*^, ^‡^	1.19 ± 1.13	0.47 ± 0.08	0.8 ± 0.06	44.27 ± 2.63^*^, ^‡^	1.17 ± 0.23	24.81 ± 2.96	4.34 ± 0.58^*^, ^‡^	0.83 ± 0.15
	RF	30.18 ± 1.28^*^, ^‡^		32.25 ± 0.48^*^, ^‡^		0.48 ± 0.08	0.81 ± 0.07	44.78 ± 2.84^*^, ^‡^	1.14 ± 0.23	24.21 ± 3.32	5.21 ± 0.68	0.83 ± 0.14
	LH	19.77 ± 1.4	0.52 ± 0.25	17.96 ± 0.	0.82 ± 0.45	0.44 ± 0.08	0.81 ± 0.06	38.19 ± 4.26	1.15 ± 0.23	21.44 ± 2.43	5.28 ± 0.67	0.9 ± 0.14
	RH	19.61 ± 1.63		18.15 ± 0.26		0.44 ± 0.08	0.81 ± 0.07	37.63 ± 3.83	1.13 ± 0.22	22.37 ± 2.3	6.35 ± 1.38	0.92 ± 0.27
Trot	LF	31.26 ± 0.47^*^, ^‡^	0.54 ± 0.39^*^	32.47 ± 0.59^*^, ^‡^	1.24 ± 0.67	0.24 ± 0.02^*^, ^‡^	1.05 ± 0.04	51.59 ± 1.43^*^, ^‡^	2.21 ± 0.18	19.79 ± 1.63	4.18 ± 1.03	0.61 ± 0.18
	RF	31.2 ± 0.64^*^, ^‡^		32.07 ± 0.39^*^, ^‡^		0.24 ± 0.02^*^, ^‡^	1.06 ± 0.05	50.93 ± 2.45^*^, ^‡^	2.24 ± 0.13	20.65 ± 1.81	4.3 ± 0.54	0.66 ± 0.16
	LH	18.89 ± 0.84	1.69 ± 0.48	17.68 ± 0.54	1.61 ± 0.99	0.21 ± 0.02	0.82 ± 0.45	43.80 ± 2.98	2.23 ± 0.19	19.02 ± 2.32	4.71 ± 1.63	0.59 ± 0.26
	RH	18.66 ± 0.23		17.78 ± 0.3		0.21 ± 0.02	1.03 ± 0.02	42.73 ± 2.41	2.16 ± 0.19	20.25 ± 2.25	5.16 ± 1.37	0.68 ± 0.19

* *Indicate a significant difference between the ipsilateral limb pairs;*

‡ *between diagonal limb pairs*.

### Control—No Dog Boots

Both PFz (%) and IFz (%) showed significantly higher values for the front limbs than for the hind limbs in both gaits (*P* = 0.000). A significant difference between the front and hind limb pairs was observed in the SI PFz (%) during trotting (higher in the hind limbs), with a *P*-value of 0.015. At trot, SPD (%) was significantly longer in the front limbs than in the ipsilateral (left front—left hind *P* = 0.022, right front—right hind *P* = 0.038) and contralateral hind limbs (left front—right hind *P* = 0.038, right front—left hind *P* = 0.019). In both gaits, the paw contact area (cm^2^) was significantly greater in the front than in the hind limbs (walk: left front—left hind *P* = 0.031, right front—right hind *P* = 0.011, left front—right hind *P* = 0.015, right front—left hind *P* = 0.024; trot: left front—left hind *P* = 0.002, right front—right hind *P* = 0.001, left front—right hind *P* = 0.000, right front—left hind *P* = 0.004). The mediolateral COP displacement (%) was significantly lower in the left front limb than in both hind limbs during walking (left front—left hind *P* = 0.046, left front—right hind *P* = 0.027). The COP area (%) did not show any significant difference in either gait when comparing individual limbs within the condition (Table 1).

### Boots on All Four Limbs

Wearing boots on all four limbs resulted in a significant increase in COP area (%) during walking in the right (*P* = 0.047) and left front limbs (*P* = 0.023) compared with the control. Similarly, a significant increase in the mediolateral COP displacement (%) during walking in the left front limb compared to the control was detected (*P* = 0.025); however, no significant difference was detected between the left front limb and both hind limbs (Table 2).

**Table 2:** Mean ± standard deviation of all parameters for the condition “boots on all four limbs.”

	**Limb**	**PFz (%)**	**SI PFz (%)**	**IFz (%)**	**SI IFz (%)**	**SPD (%)**	**SL (m)**	**PCA (cm^**2**^)**	**v (m/s)**	**COP cran-caud (%)**	**COP med-lat (%)**	**COP area (%)**
Walk	LF	29.78 ± 1.66*, ^‡^	1.12 ± 1.36	31.73 ± 1.06*, ^‡^	1.66 ± 1.41	0.51 ± 0.05	0.8 ± 0.05	44.36 ± 2.57*, ^‡^	1.08 ± 0.13	26.47 ± 4.75	5.65 ± 0.85^#^	1.12 ± 0.18^#^
	RF	29.3 ± 1.42*, ^‡^		31.99 ± 0.79*, ^‡^		0.52 ± 0.04	0.81 ± 0.06	44.68 ± 2.2*, ^‡^	1.07 ± 0.12	26.02 ± 5.82	5.72 ± 0.95	1.13 ± 0.23^#^
	LH	19.77 ± 1.4	0.86 ± 0.81	18.1 ± 0.59	1.13 ± 0.67	0.47 ± 0.04	0.8 ± 0.04	37.58 ± 4.42	1.08 ± 0.11	20.34 ± 4.04	5.94 ± 0.62	0.83 ± 0.23
	RH	20.29 ± 1.48		18.18 ± 0.69		0.47 ± 0.03	0.8 ± 0.04	37.78 ± 3.69	1.05 ± 0.1	21.32 ± 3.62	6.09 ± 1.34	0.93 ± 0.21
Trot	LF	31.26 ± 0.47*, ^‡^	1.03 ± 0.48	32.56 ± 0.94*, ^‡^	1.2 ± 1.66	0.26 ± 0.03*, ^‡^	1.01 ± 0.04	50.35 ± 2.34*, ^‡^	2.04 ± 0.27	20.45 ± 1.2	3.75 ± 0.47	0.54 ± 0.12
	RF	31.2 ± 0.64*, ^‡^		32.49 ± 0.23*, ^‡^		0.26 ± 0.02*, ^‡^	1.01 ± 0.05	50.35 ± 3.02*, ^‡^	2.09 ± 0.25	20.79 ± 2.04	4.18 ± 1.03	0.68 ± 0.27
	LH	18.89 ± 0.84	0.98 ± 1.29	17.37 ± 0.79	1.63 ± 1.05	0.21 ± 0.02	1.02 ± 0.06	41.73 ± 4.42	2.07 ± 0.27	16.77 ± 4.22	4.16 ± 1.1	0.47 ± 0.19
	RH	18.66 ± 0.23		17.58 ± 0.15		0.22 ± 0.02	1.01 ± 0.07	41.58 ± 3.7	2.04 ± 0.29	17.1 ± 4.26	5.11 ± 1.57	0.65 ± 0.08

* *Indicate a significant difference between the ipsilateral limb pairs;*

‡ *between diagonal limb pairs; ^**#**^ differences between the control and the boot wearing conditions*.

### Boots on Both Front Limbs

During walking, a significant difference in paw contact area (%) was only detected between the right hind and both front limbs (left front *P* = 0.018, right front *P* = 0.022). During walking, the COP area (%) showed a significant increase in the right front limb compared with the control, with a *P*-value of 0.054. No significant difference in the mediolateral COP displacement (%) was observed between the left front limb and both hind limbs (Table 3).

**Table 3:** Mean ± standard deviation of all parameters for the condition “boots on both front limbs.”

	**Limb**	**PFz (%)**	**SI PFz (%)**	**IFz (%)**	**SI IFz (%)**	**SPD (%)**	**SL (m)**	**PCA (cm^**2**^)**	**v (m/s)**	**COP cran-caud (%)**	**COP med-lat (%)**	**COP area (%)**
Walk	LF	30.09 ± 1.66*, ^‡^	1.23 ± 0.68	31.22 ± 0.73*, ^‡^	1.31 ± 0.89	0.49 ± 0.08	0.79 ± 0.06	44.61 ± 2.75*, ^‡^	1.09 ± 0.22	25.3 ± 4.92	4.99 ± 0.83	0.98 ± 0.23
	RF	29.91 ± 1.15*, ^‡^		31.94 ± 0.77*, ^‡^		0.5 ± 0.08	0.79 ± 0.06	44.74 ± 3.46^*^	1.09 ± 0.25	25.59 ± 4.73	5.39 ± 0.99	1.03 ± 0.15^#^
	LH	20.19 ± 1.47	1.53 ± 1.81	18.48 ± 0.79	0.59 ± 0.52	0.46 ± 0.07	0.79 ± 0.06	39.09 ± 4.94	1.09 ± 0.23	21.4 ± 4.35	5.42 ± 0.62	0.84 ± 0.22
	RH	19.81 ± 1.38		18.36 ± 0.53		0.46 ± 0.08	0.79 ± 0.06	38.14 ± 3.88	1.08 ± 0.25	21.43 ± 3.82	5.87 ± 1.4	0.92 ± 0.23
Trot	LF	30.81 ± 0.62*, ^‡^	0.58 ± 0.40	32.22 ± 1.03*, ^‡^	1.25 ± 0.98	0.25 ± 0.02*, ^‡^	0.99 ± 0.06	50.03 ± 1.96*, ^‡^	2.05 ± 0.31	20.57 ± 2.01	4.1 ± 0.94	0.6 ± 0.22
	RF	30.88 ± 0.7*, ^‡^		32.15 ± 0.51*, ^‡^		0.26 ± 0.03*, ^‡^	1.01 ± 0.07	50.2 ± 2.85*, ^‡^	2.08 ± 0.28	20.97 ± 2.83	3.99 ± 0.95	0.63 ± 0.22
	LH	19.26 ± 0.84	1.14 ± 1.10	17.87 ± 0.88	1.09 ± 0.97	0.22 ± 0.02	1.0 ± 0.05	43.34 ± 4.59	2.05 ± 0.26	18.1 ± 4.47	4.47 ± 0.93	0.58 ± 0.22
	RH	19.06 ± 0.47		17.76 ± 0.34		0.22 ± 0.02	1.01 ± 0.07	42.58 ± 3.05	2.06 ± 0.32	18.37 ± 3.77	5.79 ± 2.08	0.72 ± 0.11

* *Indicate a significant difference between the ipsilateral limb pairs;*

‡ *between diagonal limb pairs;*

# *differences between the control and the boot wearing conditions*.

### Boots on Both Hind Limbs

When boots were worn on both hind limbs at trot SI IFz (%) decreased in the hind limbs and increased in the front limbs, which led to a significant difference between the front and hind limb pairs (*P* = 0.033). A significant increase in the mediolateral COP displacement (%) in the right front limb was found during walking compared to that in the control (*P* = 0.036), which led to a significant difference between the front limbs in this condition (*P* = 0.024), but not between the left front limb and both hind limbs. Likewise, a significant increase in the right front limb occurred in the COP area (%) compared with the control (*P* = 0.012, Table 4).

**Table 4:** Mean ± standard deviation of all parameters for the condition “boots on both hind limbs.”

	**Limb**	**PFz (%)**	**SI PFz (%)**	**IFz (%)**	**SI IFz (%)**	**SPD (%)**	**SL (m)**	**PCA (cm^**2**^)**	**v (m/s)**	**COP cran-caud (%)**	**COP med-lat (%)**	**COP area (%)**
Walk	LF	30.35 ± 1.27*, ^‡^	1.08 ± 0.98	31.9 ± 1.21*, ^‡^	1.10 ± 1.61	0.51 ± 0.09*, ^‡^	0.81 ± 0.08	45.26 ± 2.88	1.1 ± 0.28	25.02 ± 3.24	4.91 ± 0.99†	0.95 ± 0.22
	RF	30.2 ± 1.11*, ^‡^		32.37 ± 0.94*, ^‡^		0.52 ± 0.08*, ^‡^	0.8 ± 0.07	46.03 ± 3.44	1.1 ± 0.25	24.69 ± 3.34	6.92 ± 1.25^#^	1.18 ± 0.19^#^
	LH	19.85 ± 1.06	1.92 ± 1.24	17.88 ± 0.63	1.51 ± 1.12	0.47 ± 0.08	0.8 ± 0.07	37.49 ± 4.26	1.08 ± 0.24	21.2 ± 4.6	6.1 ± 1.35	0.94 ± 0.25
	RH	19.6 ± 1.32		17.85 ± 0.91		0.47 ± 0.08	0.8 ± 0.08	36.96 ± 4.47	1.1 ± 0.27	21.55 ± 3.46	6.17 ± 1.53	0.95 ± 0.27
Trot	LF	30.95 ± 0.91*, ^‡^	0.93 ± 0.96	32.74 ± 1.39*, ^‡^	1.75 ± 1.03^*^,	0.26 ± 0.03*, ^‡^	1.01 ± 0.06	51.56 ± 3.23	2.05 ± 0.21	19.76 ± 2.4	4.55 ± 0.95	0.69 ± 0.26
	RF	30.97 ± 0.59*, ^‡^		32.74 ± 0.39*, ^‡^		0.26 ± 0.02*, ^‡^	0.99 ± 0.03	51.1 ± 3.75	1.95 ± 0.16	20.08 ± 2.6	4.22 ± 1.1	0.67 ± 0.23
	LH	19.14 ± 0.94	1.34 ± 0.86	17.37 ± 0.99	1.16 ± 1.21	0.21 ± 0.02	0.74 ± 0.48	42.02 ± 2.88	1.94 ± 0.15	17.74 ± 4.26	4.72 ± 1.4	0.66 ± 0.28
	RH	18.95 ± 0.35		17.35 ± 0.55		0.22 ± 0.02	1.0 ± 0.05	42.35 ± 3.31	1.98 ± 0.27	17.7 ± 3.19	5.44 ± 0.94	0.68 ± 0.07

* *Indicate a significant difference between the ipsilateral limb pairs;*

† *between contralateral limb pairs;*

‡ *between diagonal limb pairs;*

# *differences between the control and the boot wearing conditions*.

### Boot on the Left Front Limb

A decrease in PFz (%) in the left front limb was found during trotting when wearing a boot on the left front limb compared with the control (*P* = 0.022). This resulted in a significant difference between the front limbs (*P* = 0.008). Furthermore, a significant increase in PFz (%) was observed in the right hind limb (*P* = 0.025). The SI PFz (%) of the front limb pair increased significantly during trot compared with the control (*P* = 0.019). During walking and trotting, IFz (%) showed a significant difference between both front limbs in this condition (walk: *P* = 0.004, trot: *P* = 0.014), with a significant increase in the right front limb compared to the control (*P* = 0.041). At trot, SPD (%) increased in the front legs and showed a significant difference only between the right front limb and both hind limbs (*P* = 0.014) but not in comparison to the controls. The COP area (%) in the right front limb during walking increased significantly in this condition compared to that in the control (*P* = 0.013), resulting in a significant difference between the right front limb and both hind limbs (right front—left hind *P* = 0.013, right front—right hind *P* = 0.044). At both gaits for mediolateral COP displacement (%), no significant difference was observed when comparing the conditions with each other or when comparing the individual limbs within each condition (Table 5).

**Table 5:** Mean ± standard deviation of all parameters for the condition “boots on the left hind limb.”

	**Limb**	**PFz (%)**	**SI PFz (%)**	**IFz (%)**	**SI IFz (%)**	**SPD (%)**	**SL (m)**	**PCA (cm^**2**^)**	**v (m/s)**	**COP cran-caud (%)**	**COP med-lat (%)**	**COP area (%)**
Walk	LF	29.88 ± 1.89*, ^‡^	1.86 ± 1.20	30.65 ± 1.02^*^, ^†^, ^‡^	3.84 ± 2.02	0.51 ± 0.06	0.79 ± 0.07	44.03 ± 2.36*, ^‡^	1.05 ± 0.16	25.69 ± 4.7	5.3 ± 1.21	1.04 ± 0.21
	RF	30.19 ± 1.47*, ^‡^		33.09 ± 0.83*, ^‡^		0.54 ± 0.06	0.79 ± 0.05	45.16 ± 2.52*, ^‡^	1.03 ± 0.12	24.53 ± 3.97	7.24 ± 2.04	1.27 ± 0.25*, ^‡^, ^#^
	LH	20.03 ± 1.69	0.82 ± 0.73	17.96 ± 0.67	1.76 ± 1.09	0.48 ± 0.05	0.79 ± 0.06	38.06 ± 4.32	1.05 ± 0.16	21.37 ± 3.36	5.79 ± 0.67	0.82 ± 0.1
	RH	19.91 ± 1.42		18.3 ± 0.84		0.49 ± 0.05	0.78 ± 0.05	38.25 ± 3.58	1.02 ± 0.12	22.21 ± 2.58	6.1 ± 1.32	0.9 ± 0.23
Trot	LF	30.16 ± 0.69*, ^#^ ^†^, ^‡^, ^#^	2.28 ± 0.62^#^	31.39 ± 0.91*, †, ‡	2.63 ± 1.57	0.26 ± 0.03	0.99 ± 0.06	49.67 ± 1.91*, ^‡^	2.0 ± 0.26	19.21 ± 1.23	4.38 ± 0.51	0.58 ± 0.14
	RF	31.57 ± 0.52*, ^‡^		33.09 ± 0.78*, ‡, #		0.26 ± 0.02*, ^‡^	1.02 ± 0.03	51.77 ± 2.58*, ^‡^	2.06 ± 0.15	20.02 ± 1.77	4.47 ± 0.91	0.67 ± 0.11
	LH	18.93 ± 0.8	1.60 ± 0.89	17.61 ± 1.06	1.99 ± 0.82	0.22 ± 0.02	1.02 ± 0.03	42.76 ± 3.45	2.05 ± 0.17	17.65 ± 4.05	4.31 ± 1.15	0.6 ± 0.3
	RH	19.35 ± 0.46		17.91 ± 0.35		0.22 ± 0.02	1.0 ± 0.06	43.19 ± 2.92	1.98 ± 0.29	18.15 ± 2.88	5.38 ± 1.68	0.67 ± 0.09

* *Indicate a significant difference between the ipsilateral limb pairs;*

† *between contralateral limb pairs;*

‡ *between diagonal limb pairs;*

# *differences between the control and the boot wearing conditions*.

### Boot on the Right Hind Limb

At trot, SPD (%) showed only a significant difference between the left hind limb and both front limbs (front left—hind left *P* = 0.035; front right—hind left *P* = 0.046). No significant difference in the mediolateral COP displacement (%) was observed during walking or trotting when comparing the conditions or when comparing the individual limbs within each condition. Wearing a boot on the right hind limb led to a significant increase in the COP area (%) during walking in the left and right front limbs compared to the control, with a *P*-value of 0.050 (left front) and 0.053 (right front) (Table 6).

**Table 6:** Mean ± standard deviation of all parameters for the condition “boots on right hind limbs.”

	**Limb**	**PFz (%)**	**SI PFz (%)**	**IFz (%)**	**SI IFz (%)**	**SPD (%)**	**SL (m)**	**PCA (cm^**2**^)**	**v (m/s)**	**COP cran-caud (%)**	**COP med-lat (%)**	**COP area (%)**
Walk	LF	30.35 ± 1.47*, ^‡^	1.27 ± 0.79	31.92 ± 1.01*, ^‡^	1.97 ± 1.66	0.51 ± 0.09	0.81 ± 0.06	45.18 ± 2.87*, ^‡^	0.92 ± 0.48	25.29 ± 3.79	5.69 ± 1.41	1.08 ± 0.19^#^
	RF	30.34 ± 0.9^*^, ^‡^		32.41 ± 1,0^*^, ^‡^		0.52 ± 0.09	0.81 ± 0.06	45.81 ± 3.34^*^, ^‡^	0.9 ± 0.52	24.81 ± 3.73	6.79 ± 1.57	1.11 ± 0.23^#^
	LH	19.42 ± 0.91	1.83 ± 1.23	17.87 ± 0.71	0.94 ± 0.12	0.47 ± 0.08	0.81 ± 0.05	38.19 ± 5.44	0.89 ± 0.44	21.17 ± 3.68	5.7 ± 0.59	0.89 ± 0.13
	RH	19.89 ± 1.4		17.81 ± 0.45		0.47 ± 0.08	0.8 ± 0.06	36.99 ± 4.25	0.9 ± 0.52	21.71 ± 3.79	5.62 ± 1.39	0.87 ± 0.24
Trot	LF	30.93 ± 0.89^*^, ^‡^	0.97 ± 0.99	32.26 ± 1.25^*^, ^‡^	1.27 ± 1.48	0.26 ± 0.03*	1.01 ± 0.07	51.37 ± 2.71^*^, ^‡^	2.04 ± 0.28	20.45 ± 1.63	4.94 ± 2.38	0.78 ± 0.43
	RF	31.16 ± 0.73^*^, ^‡^		32.53 ± 0.15^‡^		0.26 ± 0.03^*^, ^‡^	1.02 ± 0.07	51.48 ± 3.45^*^, ^‡^	2.09 ± 0.35	19.69 ± 2.13	4.18 ± 1.05	0.57 ± 0.15
	LH	19.06 ± 0.92	1.26 ± 1.05	17.63 ± 0.71	1.61 ± 0.93	0.22 ± 0.02	0.83 ± 0.41	42.95 ± 3.71	2.07 ± 0.35	18.23 ± 4.31	4.49 ± 1.01	0.6 ± 0.23
	RH	18.84 ± 0.53		17.58 ± 0.74		0.22 ± 0.03	0.99 ± 0.06	41.86 ± 3.45	1.96 ± 0.3	17.95 ± 3.53	5.26 ± 2.3	0.68 ± 0.25

* *Indicate a significant difference between the ipsilateral limb pairs;*

‡ *between diagonal limb pairs;*

# *differences between the control and the boot wearing conditions*.

## Discussion

Dogs may need to wear paw boots for a wide variety of reasons, whether for sports or for medical purposes. It is therefore important to recognize changes in loading on the dog's legs when they are worn. The hypothesis that wearing the tested boots on single or multiple limbs results in a measurable change in the ground reaction forces of the entire limb, as well as a change in the COP area (%) and craniocaudal and mediolateral COP displacement (%) of the paw in dogs, despite prior habituation, was partially confirmed in this study.

With regard to GRF parameters, wearing a boot on the left forelimb primarily showed an effect indicating a significant redistribution of GRF toward the contralateral front limb and, in the case of PFz (%), also toward the diagonal hind limb. Interestingly, this effect was not observed when a boot was worn on only one hind limb. However, in comparison with the existing literature regarding the compensatory effects of lameness, similarities can certainly be found. For instance, a study in dogs with osteoarthrosis of the elbow joint showed a comparable redistribution of GRF evaluated on a pressure plate (23). Regarding hind lameness, research performed on pressure or force plates provides different results; for example, dogs with orthopedic diseases of one hind limb usually show an increase in GRF in the contralateral limb, and compensations to the front are rarely described (17, 34–36). Accordingly, while wearing a boot on the front limb tends to cause compensation in the dog, wearing it on the hind limb does not seem to cause any interference. This could possibly be due to the fact that the forces acting on the front limbs are generally higher than those acting on the hind limbs (24, 34, 37, 38). However, it appears that the effect no longer occurs once the animal wears the boots on both front limbs.

In comparison with the scarce literature on the subject, some differences appear regarding the ground reaction forces. Shorter and Brown (5) used a force plate evaluation and did not show any differences in PFz and IFz, but these authors performed measurements with shoes on all four paws. In addition, the authors used a two millimeter thick ethylene-vinyl acetate pad attached to the paw with a self-adhesive tape up to the carpus. Because this boot replacement extends further proximally than the tested boots and has a sole, it differs from the boots used in the present study. A recent study that used special devices on all four dog paws (ToeGrips®) observed on a pressure platform a significant decrease in PFz in both hind limbs, as well as an elongation of SPD in all limbs (27). None of the mentioned changes could be detected in this study compared to wearing boots on all four limbs during walking or trotting, which could be due to the different fitting and effect of ToeGrips® in comparison to dog boots. As there were no significant differences in paw contact area (cm^2^) between the control and when boots were worn on different limbs, it can be assumed that the tested boots fit so tightly to the paw that no change in paw contact area could be measured. Wearing these boots also had no effect on stride length. As there are a variety of boots for dogs with different profiles and sole thicknesses, further studies comparing different types of boots would be interesting.

However, we were able to record a stronger effect on the evaluated parameters of the COP area. In each of the test conditions, the COP area increased in at least one of the forelimbs and the mediolateral COP (%) of the front limbs was affected only when boots were worn on all four limbs or both hindlimbs. Interestingly, the craniocaudal displacement of the COP (%) did not change under any of the test conditions. Measurement of the COP within the paw is a fairly new method in veterinary medicine to describe biomechanical adaptations and possible compensatory mechanisms that may occur (16, 17, 19). In healthy dogs (19), all evaluated parameters had higher values in the forelimbs than in the hind limbs. This could not be confirmed in the present study for the COP area, in which both limb pairs showed comparable values. Reicher et al. (19) used a heterogeneous dog group consisting of 20 individuals to evaluate healthy dogs. Whether the differences in the results between the studies were due to a different number of subjects or a heterogeneous group composition must be investigated in subsequent studies. Nevertheless, the changes induced by the boots did not coincide with those observed in dogs with coxarthrosis (19). Mediolateral displacement (%) did not increase in the hind limbs, but did in the forelimbs when a boot was worn on one hind limb, a situation reversed in dogs with coxarthrosis. Compared to dogs with cubarthrosis (16, 19), changes in the front limbs could also be observed; however, in the latter, the craniocaudal COP (%) on the contralateral front limb and the COP area (%) of the hind limbs increased, whereas in dogs that wore boots on one or both front limbs, the COP area (%) of the front limbs increased. An increase in COP values is generally interpreted in the literature as a sign of reduced stability (17, 39). Likewise, changes in COP parameters can also be considered in terms of biomechanical adaptations. In dogs with unilateral elbow joint dysplasia, Lopez et al. (18) described that limb COP path in lame limbs is shortened and compared with the contralateral limb cranialized, due to a larger caudal margin (which describes the distance between the most caudal limit of the paw print and the most caudal limit of the limb COP path). The authors explained this by a shortened swing phase and reduced extension, which ultimately leads to incorrect load takeover of the metacarpal pad during landing. Because the caudal margin was not evaluated in our study, further studies should investigate the extent to which this value is influenced by the wearing dog boots. The same authors also describe in their study an increased mediolateral deviation of the COP in the non-lame limb, which was interpreted as a result of an increased pad deformation caused by the increased weight bearing. The results of our study do not show comparable results for this parameter, as no changes in GRFs were observed in those conditions in which mediolateral deviation of the COP was increased. Finally, Lopez et al. detected increased values of COP area in a statokinesiogram, which they interpreted as an indication of increased instability. Also in our study, significant changes of the COP area in the front limb area were shown, which could be interpreted as an indication that the wearing of boots leads to a certain increased instability, even though the dogs have been previously habituated to wearing the boots. A possible explanation could be that sensory stimuli may be partially lost because of the rubber layer between the paw and ground. In humans, sensory input through the sole of the foot influences postural control (40, 41). The absence of plantar cutaneous sensation has also been shown to affect COP parameters when the postural control system is challenged (42).

A limiting factor of this study is the relatively small number of subjects, although this was partially compensated by the use of the same breed. Nevertheless, further studies with more animals, especially different breeds, will be necessary to clarify the effects of the tested boots on GRF and COP. Whether the thickness of the sole, stability of the shoe, and size of the contact area of the shoe with the ground lead to varying results in ground reaction forces as well as COP parameters requires further investigation. In humans, it is assumed that the cushion and thickness of the sole have an impact on the loading rate of peak GRF, being smaller when wearing shoes compared to flip-flops and sandals, and being barefoot (13). In further studies on the topic, the combination “boots on a diagonal limb pair,” which was not measured in this work, should also be tested.

In summary, this study found small changes in the GRF between wearing boots and walking without boots, but signs of reduced stability between paw and ground during the stance phase of the front legs, which should be considered when using these dog boots. Furthermore, it should be mentioned that the manufacturer warns against unsupervised use and prolonged wearing of the boots^2^. Whether long-term use will cause deviations in limb loading with further effects on the orthopedic health of dogs needs to be explored in further studies. It should also be mentioned that paw boots on only one limb are mostly used for medical reasons and therefore only for a limited period of time, for instance, until a wound has healed. The question remains as to whether these deviations in the evaluated parameters while wearing a boot actually have an effect on further orthopedic health issues.

## Data Availability Statement

The original contributions presented in the study are included in the article/supplementary material, further inquiries can be directed to the corresponding author/s.

## Ethics Statement

The animal study was reviewed and approved by Ethics and Animal Welfare Committee, University of Veterinary Medicine, Vienna, Austria. Written informed consent was obtained from the owners for the participation of their animals in this study.

## Author Contributions

BR and BBo contributed to the conception and design of the study. BR and BBi performed the measurements and data evaluation. AT performed the statistical analysis. BBi wrote the draft of the paper. BR and BBo wrote sections of the manuscript. All authors contributed to manuscript revision, read, and approved the submitted version.

## Conflict of Interest

The authors declare that the research was conducted in the absence of any commercial or financial relationships that could be construed as a potential conflict of interest.

## Publisher's Note

All claims expressed in this article are solely those of the authors and do not necessarily represent those of their affiliated organizations, or those of the publisher, the editors and the reviewers. Any product that may be evaluated in this article, or claim that may be made by its manufacturer, is not guaranteed or endorsed by the publisher.

## Footnotes

^1^Pawz Rubber dog boots. https://www.pawzdogboots.com/pawz-boots/ (accessed February 10, 2021).

^2^Pawz. Pawz Large Size dog boots. https://pawzdogboots.com/product/large-size-rubber-boots/ (accessed February 10, 2021).
